# The significance of Epstein Barr Virus (EBV) & DNA Topoisomerase II alpha (DNA-Topo II alpha) immunoreactivity in normal oral mucosa, Oral Epithelial Dysplasia (OED) and Oral Squamous Cell Carcinoma (OSCC)

**DOI:** 10.1186/1746-1596-3-45

**Published:** 2008-11-20

**Authors:** Ali A Shamaa, Manal M Zyada, Mathias Wagner, Sally S Awad, Mohamed M Osman, Ali A Abdel Azeem

**Affiliations:** 1 Oral Biology Department, Faculty of Dentistry, Minia University, Minia, Egypt; 2 Oral Pathology Department, Faculty of Dentistry, Mansoura University, Mansoura, Egypt; 3 General Pathology Department, Saarland University, Saarland, Germany; 4 Oral Surgery Department, Faculty of Dentistry, Mansoura University, Mansoura, Egypt; 5 Oral Surgery Department, Faculty of Dentistry, Misr International University, Cairo, Egypt

## Abstract

**Background:**

Head and neck cancer including oral cancer is considered to develop by accumulated genetic alterations and the major pathway is cancerization from lesions such as intraepithelial dysplasia in oral leukoplakia and erythroplakia. The relationship of proliferation markers with the grading of dysplasia is uncertain. The involvement of EBV in oral carcinogenesis is not fully understood.

**Aim:**

The present study was designed to investigate the role of EBV and DNA Topoisomerase II∝ (DNA-Topo II∝) during oral carcinogenesis and to examine the prognostic significance of these protein expressions in OSCCs.

**Methods:**

Using specific antibodies for EBV and DNA-Topo II∝, we examined protein expressions in archival lesion tissues from 16 patients with oral epithelial dysplasia, 22 oral squamous cell carcinoma and 20 normal oral mucosa by immunohistochemistry. Clinical information was obtained through the computerized retrospective database from the tumor registry.

**Results:**

DNA-Topo II∝ was expressed in all examined specimens. Analysis of Variance ANOVA revealed highly significant difference (P < 0.01) in young aged labial tissues and significant (P ≤ 0.05) in gingival and not significant (P > 0.05) in inferior surface of tongue and in hard palatal tissues. Significant differences were observed between OEDs and NSE (P < 0.001) and SCCs and controls (P < 0.001), also, significant differences could be observed between SCCs and OEDs. DNA-Topo II∝ expression was significantly higher in tumors of low differentiation versus tumors of moderate and high differentiation (P < 0.001), DNA-Topo II∝ expression was correlated with age, tumor size, tumor stage, node metastasis and tumor differentiation, but not with gender and tumor site. None of normal squamous epithelium (NSE) expressed EBV. Heterogenous reactivity for EBV was observed through the series of dysplasia and squamous cell carcinoma. Its expression increased progressively with lymph node metastasis and low tumor differentiation, but no significant association could be observed with other clinicopathological parameters. EBV protein expression was increased with elevated Topo II-∝ LI in OEDs and OSCCs. A tendency to positive correlation between EBV and Topo II∝ expression was observed in OEDs but not in OSCCs.

**Conclusion:**

EBV and DNA Topo II-αLI expression are possible indicators in oral carcinogenesis and may be valuable diagnostic and prognostic indices in oral carcinoma.

## Background

Oral carcinogenesis is generally considered to be a molecular and histologic multistep process that includes activation of oncogenes, inactivation of tumor suppressor genes and involvement of viral genes [1,2]. The histologic features are predominantly caused by alteration of cell kinetics in the proliferative pool of the epithelium, expressed as increased growth fraction and cell division rate. This alteration determines the transformation of normal oral epithelium into a malignant tumor [3]. According to this hypothesis, the steps of the transformation from normal epithelium to carcinoma are low grade and high-grade oral intraepithelial neoplasias (OINs). These dysplastic alterations are considered to be the precursory steps of the invasive squamous cell carcinoma [4].

The presence and severity of dysplasia are often regarded as an indicator of the risk status of a precancerous lesion [5]. Severe dysplasia indicates a very high risk of the subsequent development of cancer [6]. However, Lind reported that the grading of dysplasia was not proportional to the risk of independent transformation [7].

The question arises as to what can replace the routine histological reporting considered as the gold standard for assessing the risk of a potentially malignant oral lesion [3]. The search for alterations in molecular and genetic characteristics has so far not yielded predictive risk markers to assess the malignant potential of oral dysplastic lesions [8]. Among an array of genetic aberrations reported both in oral precancer and in squamous cell carcinoma (e.g. p35, p16/MTS1, and cyclin D), none has been shown to be sufficient or necessary for transformation of oral keratinocytes. Lack of clearly defined gate-keeping genes for this site has hampered progress in identifying early biomarkers of progression. Of the available biomarkers [9], one would expect those identifying genomic status and cell proliferation to correspond closely to the cellular and tissue changes observed in dysplasia [10].

Analysis of the cell kinetics of cancer cells in situ for example, by mitotic counts, DNA analysis, or Ki-67 antigen expression is used increasingly to evaluate the prognosis and/or biological behavior of various human malignancies DNA Topoisomerase II (Topo II) is thought to be one of these cell cycle related proteins, and Topoisomerase IIα (Topo IIα), one of its isoforms, has been shown to play an important role in the cell cycle through catalyzing the topological isomerisation of DNA by passing one strand of DNA through a reversible break in a second DNA strand [11]. Dysregulation or qualitative alterations of Topo IIα expression in the cell cycle are being reported in both normal tissues and various human neoplasms' [12-14]. The sensitivity or resistance of a malignant cell to several antitumour drugs known as "Topo II poisons" is quantitatively dependent on the cellular content of Topo II [15].

Although considerable insight has been gained into Epstein Barr virus (EBV) as an important etiologic factor in a variety of diseases, benign and malignant disorders [16] e.g. extra-hepatic biliary atresia [17], infectious mononucleosis [18], Burkitt' s lymphoma [19], oral hairy leukoplakia [20], salivary gland tumors [21] and nasopharyngeal carcinoma (NPC) [22], virtually little is known about the possible role of viruses and their interactions with genes [23]. EBV is a herpes virus; it is clearly associated with Burkitt' s lymphoma [19] and anaplastic carcinoma [24] and the oncogenicity is not in doubt. However, EBV-DNA antigens have not been demonstrated in carcinomas from other sites of the head & neck or cell lines [24-27]. This association has proved to be consistent regardless of the degree of differentiation of the neoplasm and extent of lymphocytic infiltration [28]. EBV can be considered a possible candidate for participation in the process of carcinogenesis for several reasons. Firstly, oral squamous epithelium can potentially support EBV persistence as exemplified by oral hairy leukoplakia. Secondly, nasopharyngeal carcinoma, also a squamous cell carcinoma, harbors EBV DNA and viral transcripts [29]. However, only limited information is available on EBV expression in head and neck including oral cancer.

In the present study, we examined immunohistochemically the expression of EBV and DNA Topoisomerase (DNA-Topo) II∝ in normal keratinized & non keratinized oral mucosa and also, to compare their expressions in oral epithelial dysplasias (OEDs) and oral squamous cell carcinomas (OSCC) with the normal oral mucosa to determine whether their expressions have the potentiality to be used as a biomarkers in the study and management of premalignant and malignant epithelial lesions.

## Methods

Paraffin-embedded, formalin-fixed tissue blocks were retrieved from archival files of Oral pathology & general pathology Departments, Faculty of Dentistry & Medicine, Mansoura and Minia Universities between November 1997 and April 2006. Thirty eight patients (12 women and 26 men) treated for primary SCC of the oral cavity and premalignant oral lesions were selected for the study. None of patients received chemotherapy or radiotherapy before operation. All diagnoses were revised, and the lesions were classified according to the World Health Organization (1997) [30]. Histologic typing of the cancer and precancer of the oral mucosa. They included 16 oral epithelial dysplasia (OED) {7 mild dysphasia with the age range of 20–64 years, 5 moderate dysplasia with the age range of 30–52 years, 4 severe dysplasia with the age of 22–71 years} and 22 oral squamous cell carcinomas (OSCCs) with the age of 24–71 years. Specimens of OED were obtained from the buccal mucosa (11 cases) and floor of the mouth (5 cases) while 22 specimens of OSCC were obtained, five from the tongue, 3 from the gingiva, 2 from the floor of the mouth, 9 from the buccal mucosa and 3 from the hard palate. Twenty-two oral squamous cell carcinoma cases were classified according to the TNM classification of the International Union Against Cancer (UICC, 2002) [31]. 22 in T categories, 8 patients were in T1, 10 in T2, 4 in T3. In N category, 9 patients were in N0, 11 in N1 and 2 in N2. Of the 22 SCCs, 5 were Stage I, 4 were Stage II, 11 were Stage III, and 2 were Stage IV. Histopathologically, 10 were well differentiated SCC, 8 were moderate differentiated SCC, 4 were poorly differentiated SCC corresponding to Grades I, II, and III respectively.

Twenty tissue specimens of different normal oral tissues (gingiva, hard palate, lip and inferior surface of tongue as specialized mucosa) were used as control. The chosen specimens were retrieved from Oral surgery Department, Faculty of Dentistry, Mansoura and Minia Universities. The specimens were classified according to their different locations and ages. The specimens are divided according to the age into two groups: first group from 20 to 40 years, early and late adolescence. The second group from 40 to 75 years.

### I-Clinical study

The clinical data were collected retrospectively through the computerized retrospective database from the lesion registry, regarding age, sex, site, tumor size and lymph node metastasis.

### II-Histological study

5 μm thick sections were prepared for (a) routine haematoxylin and eosin stain for histological examinations and (b) on positively charged glass slides (Optiplus) Biogenex, San Ramon, California, USA., for immunostaining procedures.

### III-Immunohistochemical study

Two sections were obtained from each case, one for positive test slide and the other for negative control (by omitting the primary antibody and non-immune serum is used instead of primary antibodies). Immunohistochemical staining was carried out using labeled streptavidin-biotin peroxidase complex technique Enzinger FM and Weiss SW 1988[32] (DAKO LSAB +Kit, HRP) DAKO-Dakopatts, Glostrup, Denmark(code no.k0679). After dewaxing and rehydration, sections were incubated for 5 minutes at 37°C in 0.05% protease type XXIV Sigma in phosphate buffered saline (PBS). This was followed by endogenous peroxidase block using 0.3% menthanolic H2O2. Sections were then washed in PBS, and incubated for 5 minutes in 1% Bovine Serum Albumin (BSA) to reduce non specific binding. The monoclonal antibodies for EBV CS1-4 (Dakopatts, diluted at 1:50) that recognizes EBV-encoded LMP1 and mouse anti-human Topoisomerase II α protein (DAKO, clone: Ki-S1, isotype: IgG2a) were used. The bottle contains 1 ml of Topo II α antibody provided in liquid form as purified IgG diluted in 0.05 M Tris/HCL, 15 mM NaN, pH 7.2, 1% bovine serum albumin (BSA). (Bottle no. 2) was applied to 1:80 dilutions in 1% BSA in PBS. After overnight incubation at 4°C, sections were washed and treated with anti-rabbit, anti-mouse and anti-goat Ig in phosphate buffered saline (PBS) for 30 minutes at room temperature, then followed by streptavidin peroxidase conjugate 1:300 at 37°c for 20 min. Sections were then washed in PBS, visualized with diamino-benzidene H_2_O_2 _(DAB) and counterstained with Mayer's hematoxyline. Positive and negative controls were included. For negative control slide, one vial (3 ml) of non-immune serum or immunoglobulins in PSA with 0.09% sodium azide was used.

### Immunohistochemical evaluation

The immunoreactivity of each specimen was evaluated by Light microscope to assess the intensity of EBV expression, graded according to a 5-grade scale, and the labeling index (LI) of DNA-Topo II∝. The percentage of EBV positive tumor cells showing nuclear and cytoplasmic staining was determined by counting a total of 2000 Cells in 10 randomly selected fields of each section examined at 400 magnifications. EBV expression was then classified as grade 0 (negative expression); grade 1 (low expression), 1–25% of tumor cells positive; grade 2 (intermediate expression), 26–50% positive; grade 3 (high expression), 51–75% positive; or grade 4 (high expression), 76% or more positive.

To evaluate the DNA-Topo II ∝ LI, cell nuclei showing intense homogeneous brown color or granular staining were counted as positive in a similar manner as described for EBV expression. The LI was defined by the percentage of positively stained cell nuclei and the intensity was expressed as 1+ to 4+.

### Statistical analysis

It was performed using SPPS computer program v.11.0 Full. One-way ANOVA (analysis of variance) test, Independent t-test, Spearman's rank correlation coefficient were used for statistical analysis. Bonferroni test was used for post hoc test. A p value less than 0.05 was regarded as statistically significant.

## Results

### EBV expression and Topo Π- α LI in different normal oral tissues

**Figure 1:**
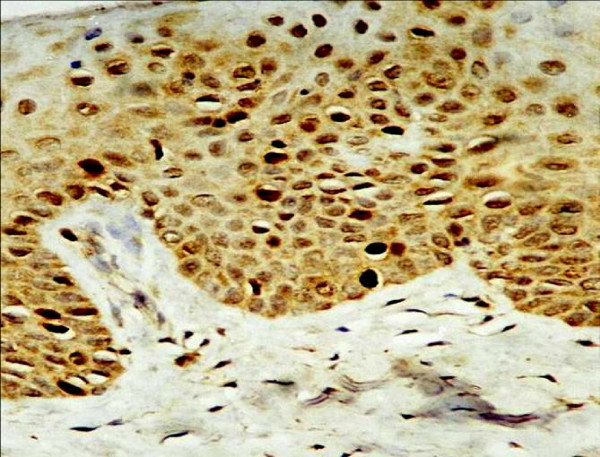
Strong Topo IIα positivity is seen mainly in basal cells with scattered distribution in human lip of old age group (× 250).

**Figure 2:**
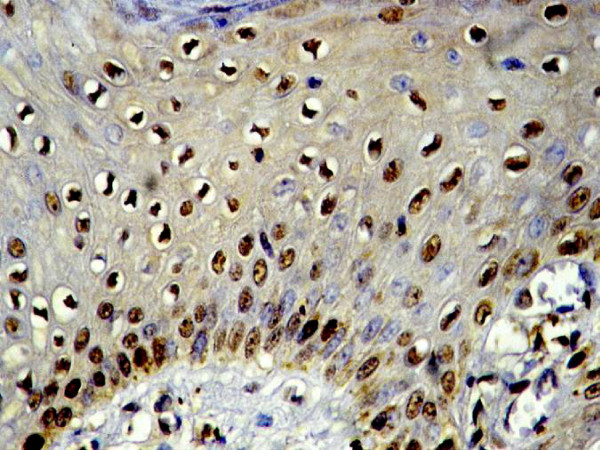
Strong Topo IIα positivity is seen in human gingiva of young age group (× 250).

In normal squamous epithelium (NSE), EBV expression was negative, and Topo II∝ LI was low (5.4%). Topo II-∝ was mainly observed in the basal and parabasal cell layers (Fig 1, 2) ranging from 1.9% to 9.7% with a mean of 5.4%.

The results of the Analysis of variance (ANOVA) which was used to test Topo II-α LI in young aged groups of gingival, labial, hard palate, inferior surface of tongue tissue were listed in (Table 1). These results revealed highly significant difference between young aged labial tissues versus old aged groups (P < 0.01), significant (P ≤ 0.05) in gingival tissues and not significant (P > 0.05) in hard palate and inferior surface of tongue tissues.

**Table 1:** showed mean of Topo II-α positive cell in Lip, Gingiva, Tongue and Hard palate in Young and Old aged group of Human different tissue.

Mean ± SE				
Statistical Profile	Lip(a)	Gingiva(b)' Tongue (c)	Inferior S. of	Hard palate (c)

Young aged group	7.68 ± 0.31	8.59 ± 1.05	5.00 ± 2.12	5.00 ± 0.71

Old aged group	2.35 ± 0.64	4.63 ± 0.91	2.85 ± 0.07	4.60 ± 1.08

F value	7.350	0.097	2.4E^+^17	o.771

P value	0.002	0.01	1.000	1.000

Significant	A = ***	b = **	C = *	c = *

(a) = *** P < 0.01 highly significant; (b) = ** P ≤ 0.05 significant; (c) = * P > 0.05 NS = Not significant.

### EBV expression and Topo ∏-∝ LI in oral epithelial dysplasia

**Figure 3:**
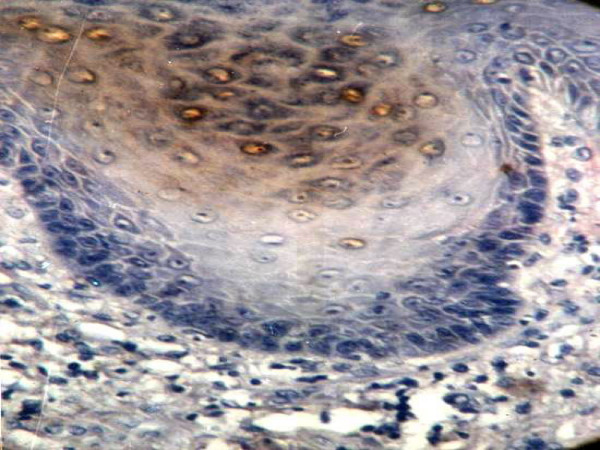
Mild dysplasia shows existence of EBV in the prickle cells(× 400).

**Figure 4:**
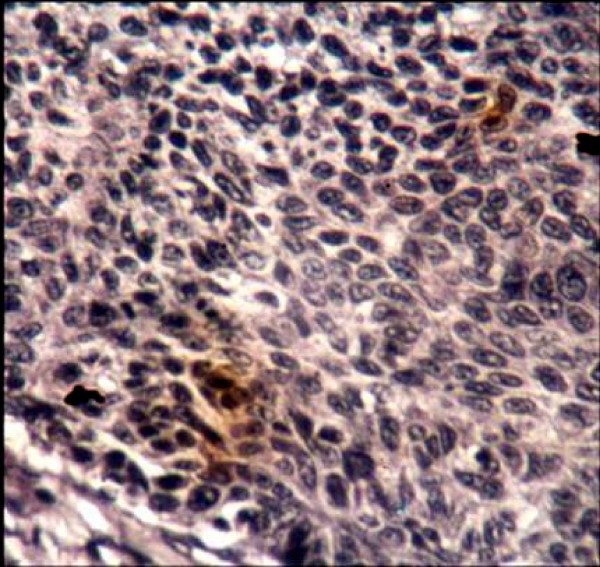
Moderate EBV positivity in moderate epithelial dysplasia (× 400).

In oral epithelial dysplasia, ten out of the sixteen cases (62.5%) were immunoreactive for EBV. Four cases (25%) showed strong (+++) reaction, Four cases (25%) were moderate (++) and two (12.5%) were mild (+), the positive immunosignals appeared as dark golden brown, focal or diffuse staining within the cytoplasm and/or nucleus corresponding to grades 1–4 in some dysplastic cells. The reaction indicating the existence of EBV was detected in the positive cases with variable degrees of intensity and distribution. In mild dysplasia, strong positively stained cells were concentrated in the prickle cell layer of the surface epithelium while the basal cells were weakly stained (Fig. 3). In some cases of moderate dysplasia, immunohistochemically detectable EBV was confined to the nucleus (Fig. 4). Few cases of severe epithelial dysplasia exhibited both nuclear and cytoplasmic reaction (total cell reactivity).

**Figure 5:**
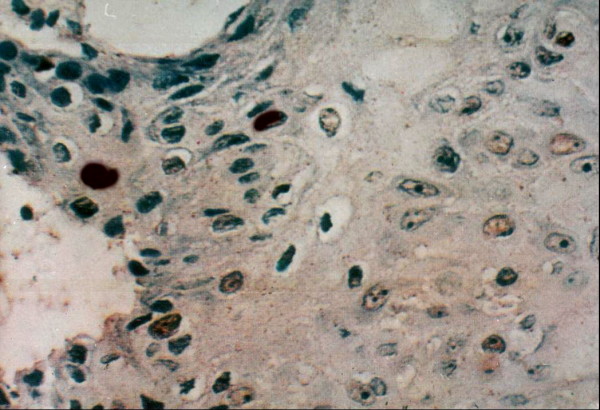
Moderate to intense EBV positivity in keratinized cells and central malignant cells in well to moderate differentiated SCC (× 400).

The Topo II-∝ expression was detected in all cases studied. The staining was usually uniform over nuclei or granular, though some variations in staining intensity and staining pattern were observed (Fig. 5). The staining was observed in basal and parabasal cell layers.

Table 2 shows that the highest mean of EBV and Topo II-α was detected in severe epithelial dysplasia (48.75 ± 33.51, 17.98 ± 1.51% respectively). A highly significant difference between this dysplasia and control was observed (t = -7.193,-10.78 respectively). On the other hand, the lowest mean of EBV and Topo II-α was found in mild epithelial dysplasia (13.43 ± 13.24, 12.27 ± 2.17% respectively). Statistical analysis demonstrated significant difference between this epithelial dysplasia change and its control (t = -4.714, -7.136 respectively). Moreover, the overall mean of EBV and Topo II-α of oral epithelial dysplasia (25.56 ± 25.38, 14.08 ± 2.99% respectively) was significantly higher compared with control (t = -4.521, -10.056 respectively).

**Table 2:** Expression of EBV and Topo II∝ in the normal oral tissues, oral epithelial dysplasia and oral squamous cell carcinomas

HISTOLOGY	Number of cases	EBV expression(%)^(a)^	P-value	Topo II-∝ LI (%)^(b)^	P-value
NSE	20	0.000 ± 0.000	d	5.35 ± 2.22	c
OED	16	25.6 ± 25.38		14.08 ± 2.99	
Mild	7	13.43 ± 13.24	NS	12.27 ± 2.17	NS
Moderate	5	24.00 ± 22.62		13.50 ± 1.86	
Severe	4	48.75 ±		17.98 ± 1.51	
OSCC	22	41.79 ± 29.31		27.56 ± 7.49	
W-scc	10	36.09 ± 22.37		22.40 ± 3.19	
M-scc	8	41.15 ± 33.18	NS	28.54 ± 4.84	e
P-scc	4	57.33 ± 38.91		39.00 ± 6.45	

NSE, normal squamous epithelium; OED, OraLepithelial dysplasia; OSCC, oral squamous cell carcinoma; NS, not significant.

^a ^mean ± standered deviation of EBV

^b ^mean ± standered deviation of Topo II-α LI.

^c ^NSE vs. OED P < 0.001;NSE vs. SCC, P < 0.001; OED vs. SCC, P = 0.083.

^d^NSE vs. OED P < 0.001;NSE vs. SCC, P < 0.001; OED vs. SCC, P < 0.001.

^e^W-SCC vs. M-SCC, P = 0.008; W-SCC 1 vs.P-SCC, P = 0.001; W-SCC vs. M-SCC +P-SCC, P < 0.001.

### EBV expression and Topo II-∝ ILI in oral squamous cell carcinoma

Eighteen out of the twenty-two cases (81.8%) of OSCC showed immunopositivity for EBV. Among these eighteen cases, ten cases showed strong (+++) reaction, while the remaining eight cases revealed weak (+) to moderate (+++) reaction.

**Figure 6:**
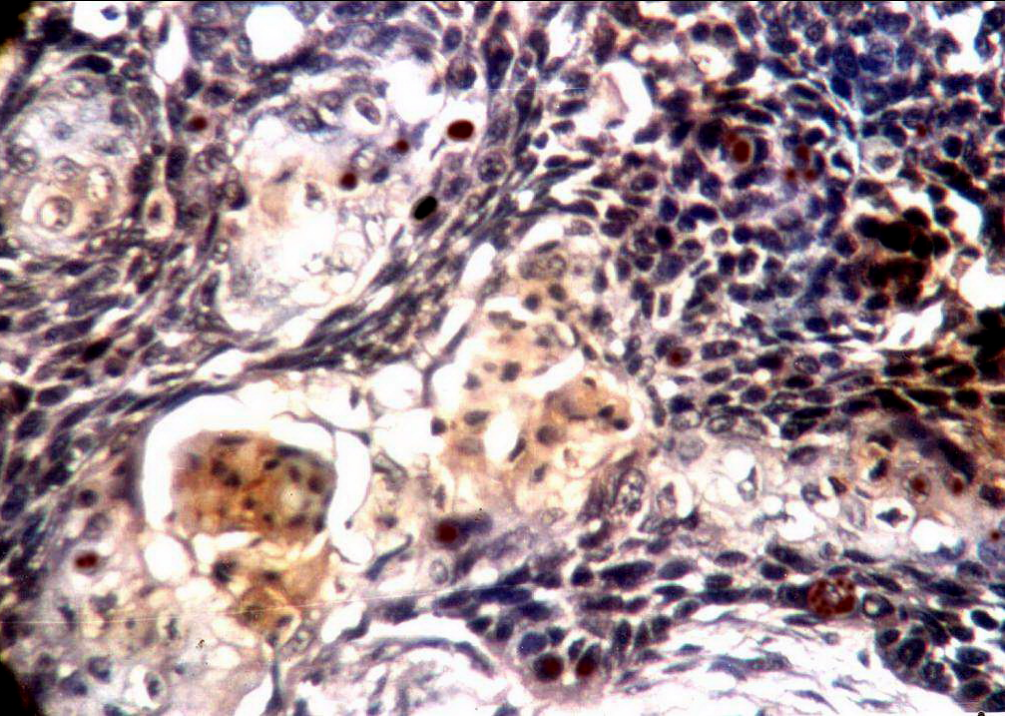
Moderate Topo IIα positivity is observed mainly in dysplastic cells of severe epithelial dysplasia (× 400).

The strong positively stained cells were concentrated in the central malignant cells while peripheral cells of epithelial pearls and islands were weakly stained. Immunoreactive EBV was detectable within the keratinized cells (Fig. 6). A positive cytoplasmic reaction was noted in seven cases while the other eleven cases demonstrated both nuclear and cytoplasmic reaction. The expression rate was 36.09% in well-differentiated SCC, 41.15% in moderate-differentiated SCC and 57.33% in poorly differentiated SCC (Table 2).

**Figure 7:**
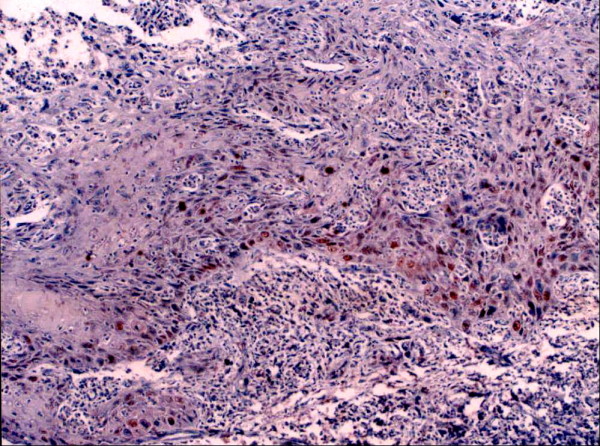
Moderate Topo IIα positivity is seen in moderately differentiated SCC (× 100).

Topo II-∝ staining was seen as a granular or distinct, diffuse nuclear pattern in basal and suprabasal cell layers (Fig. 7, 8 &9). Topo II-∝ expression was detected in all studied cases and the Topo ∏-∝ LI was 22.40% in well-differentiated SCC, 28.54% in moderate-differentiated SCC and 39.00% in poorly differentiated SCC (Table 3).

**Figure 8:**
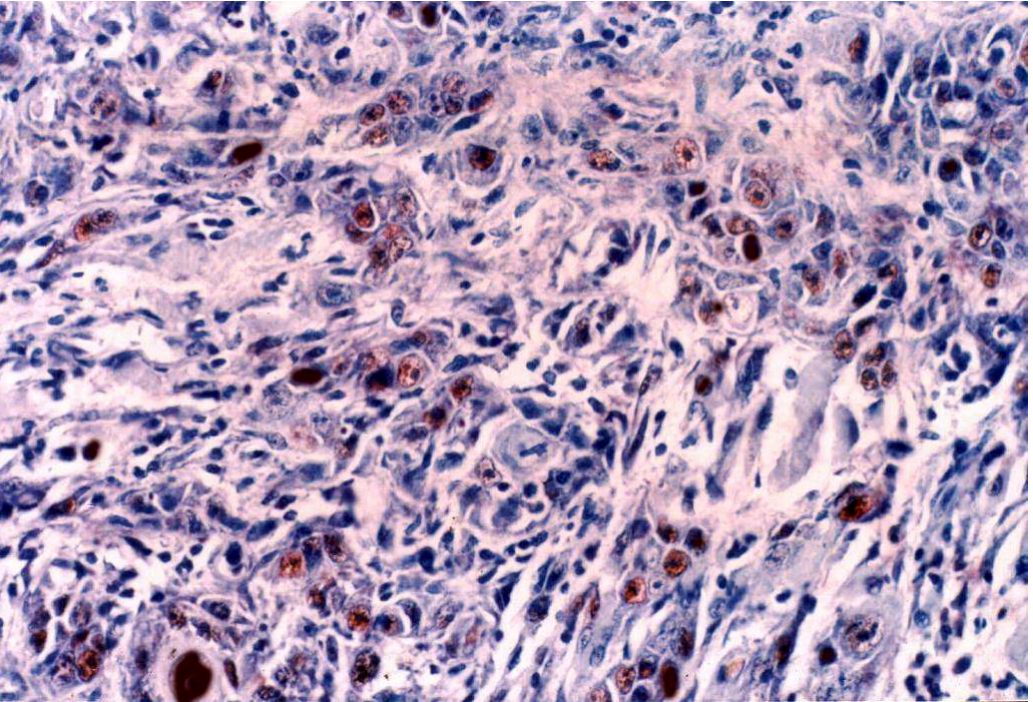
Strong Topo IIα positivity is seen in poorly differentiated SCC (× 250).

**Figure 9:**
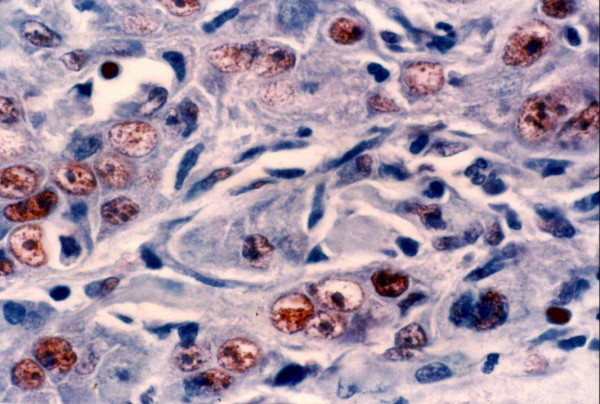
**Higher magnification appearance of Topo IIα immunoreactive cells**. Reaction is granular in most cells (× 400).

**Table 3:** Expression of EBV and Topo II∝ in relation to clinicopathological parameters in oral squamous cell carcinoma

Parameter	Total n.	EBV	P-value	Topo II-∝	P-value
Stage T	22				
T1	8	29.24 ± 46.00	0.191	24.68 ± 5.96	0,038
T2	10	50.36 ± 30.71		26.38 ± 5.22	
T3	4	45.48 ± 37.79		36.78 ± 9.39	

N stage	22				
N-	9	23.59 ± 23.71	0.009	23.04 ± 3.95	0.002
N+	13	54.39 ± 26.62		30.84 ± 7.80	

Differentiation	22				
Well-diff.	10	36.09 ± 22.37	0.208	22.40 ± 3.19	
Moderate-diff	8	41.15 ± 33.18		28.54 ± 4.84	0.000
Poorly-diff.	4	57.33 ± 38.91		39.00 ± 6.45	

### Relationship between EBV and Topo II-∝ immunostaining and Clinicopathologic Characteristics in OSCC

Expression of EBV and DNA-Topo II∝ were evaluated with respect to various prognostic factors including tumor size (T), lymph node status (N), and stage. As almost all of cancerous lesions showed high expression of grades 3 and 4 in EBV, 2+ and 3+ in DNA-Topo II∝ LI, distributions of intensity of their expression were examined for T, N, M categories and stage. Consequently, the intensity increased as T, N, M and stage advanced (Table 3). It was observed that patients with higher incidence of lymph node metastasis had high both Topo II-∝ LI and EBV expression. The significant correlation between the presence of lymph nodes metastasis and both Topo II-∝ LI and EBV was noted (Spearman's correlation coefficients method r = 0.635; P = 0.002, r = 0.541; P = 0.009 respectively). Furthermore, the one way analysis of variances method (ANOVA) showed significant differences between (N-) and (N+) groups (F= 17.54, 4.88 respectively).

Topo II-∝ L indices and EBV expression were compared to tumor status. Analysis of variance showed that there were significant differences among the groups for Topo II-∝ LI (F = 5.262). Significant differences were demonstrated between (T_1_) and both (T2) & (T_3_) groups. Also Topo II-∝ L indices correlated significantly with T status of tumor (r = 0.445; P = 0.038). However, no Significant correlation was observed between EBV and T status of tumor (r = 0.290; P = 0.191).

The highest Topo II-∝ LI and EBV were found in stage IV stage case. This case diagnosed histologically as poorly differentiated squamous cell carcinoma. The lowest Topo II-∝ LI was found in two cases, one case was in stage I and the other in stage III, however, both were diagnosed as well differentiated SCC. Both cases had low EBV.

Comparison of means of Topo II-∝ LI by analysis of variance (ANOVA) showed significant difference among the groups (F = 11.10). It was found that a significant difference could be noted between stage I and stage IV groups (Bonferroni test at significance level 0.05). Moreover, the Spearman's correlation coefficients method showed a significant correlation between the clinical staging of tumors and both Topo II-∝ Indices and EBV (r = 0.630; P = 0.002 r = 0.590; P = 0.004 respectively).

Indeed, the analysis by ANOVA test did not provide us with statistical differences between expression of EBV and both of tumor site and age in OEDs and OSCCs. However, there was a significant difference between male and female in relation to EBV in OSCCs (F = 8.566) in which EBV showed high expression in male. While, there was a significant difference between young and old age in relation to DNA-Topo IIα LI in OSCCs (F = 18.099) in which DNA-Topo IIα LI showed high expression in young age. Furthermore, no significant correlation between DNA-Topo IIα LI and both of tumor site and gender in OEDs and OSCCs.

### Correlations between expression of EBV and Topo ∏-∝

When correlation between EBV and Topo ∏-∝ was evaluated in OSCC and OED. There was a significant correlation between them (r = 0.491; P = 0.054) observed in OEDs not in OSCCs (r = 0.081; P = .720).

## Discussion

It is well established that oral carcinogenesis derives from progression of preinvasive neoplastic lesions that are characterized by grade specific morphological alterations [33]. Viruses have been implicated in malignant neoplasia of squamous epithelia so it is conceivable that they might contribute aetiologically in some cases of oral carcinoma [1].

Although overexpression of EBV is considered to contribute to oral carcinogenesis, there is few literatures supporting this hypothesis [34,35] and several reports described the up-regulation of EBV expression in dysplasia and carcinoma of the head and neck cancer [34,36]. Therefore, the present study was designed to investigate the immunohistochemical expression of EBV in the 38 cases of oral epithelial dysplasia and oral squamous cell carcinoma and thereby to elucidate the involvement of EBV in oral carcinogenesis. In the present study, pattern of EBV immunostaining in the normal control sections revealed negative results. This coincides with the results of Talacko et al [37] and Shimakage et al [38] who found no detectable EBV DNA in oral mucosa of normal individuals using in situ hybridization technique. They suggested that EBV replicates upon entry into the oral terminally differentiated keratinocytes rather than remaining latent.

Our study showed that 10 out 16 cases (62.5%) of epithelial dysplasia were positive for anti-EBV antibody. This is in agreement with Cruz and co-workers [36] who reported the presence of EBV in 77.8% of premalignant lesions. In contrast, Naher et al [39] and Horiuchi et al [40] found that EBV genome was detected in 8.6% and 5.3% of their studied oral leukoplakia cases respectively.

An interesting finding was noted in the present study. The EBV genome was detected in the middle and upper layers of squamous epithelium more than in the basal cell layer. This result agrees with Greenspan [20] and Sandvei [41] who demonstrated the presence of EBV receptors in the prickle layer of normal oral epithelium and oral hairy leukoplakia.

In the current study, 18 out of the 22 (81.8%) examined squamous cell carcinomas were positive for anti-EBV antibody. This is in agreement with Mao and Smith [16] and Horiuchi et al [40] who showed that 35% and 33.3% of their examined squamous cell carcinoma cases respectively were infected with EBV. They stated that the positively rate was higher in malignant lesions than in benign ones and agreed that EBV had a role in carcinogenesis; however, it may exist in cancer cells as a passenger. A higher percentage was noticed by Van Rensburg [34], Cruz and Co-workers [36]. They observed EBV in 100% of the oral squamous cell carcinomas, reflecting difference in analytical methods as they used PCR which is a highly sensitive technique.

Contradictory to our results, Talacko et al [37] using insitu-hybridization demonstrated that EBV DNA was not present in the neoplastic cells of oral squamous cell carcinoma. They concluded that there was no evidence for a role of EBV in the process of malignant transformation of oral mucosa in immunocompetent individuals.

In the present work, immunoreactive EBV was detected within the keratin pearls and in the central malignant cells of squamous cell carcinoma. The pattern of staining was nuclear, perinuclear, cytoplasmic as well as total cell reactivity. This is in agreement with the findings of Greenspan [20], Horiuchi et al [40], and Sandvej [41] who confirmed the presence of EBV in a similar pattern of reaction.

Thus in this study, the presence of EBV proteins was demonstrated in (73.6%) of all our studied cases while negative results were observed in the control specimens. This result implies a possible direct relation between EBV infection and epithelial dysplasia as well as squamous cell carcinoma. Accordingly, our study appears to support the view of others concerning the role of EBV in carcinogenesis [34,35].

In the present study, cancerous lesions of the oral mucosa expressed EBV more prominent than did oral epithelial dysplasia lesions. EBV expression was increased as tumor differentiation was decreased.

In our study, we found that increased age did not enhance EBV prevalence. Thus, the age does not seem to be a risk factor for EBV infection. This result was in accordance with other reports [35].

The relationship of proliferation markers with the grading of dysplasia is uncertain, and the present investigation is an attempt to remedy this. Recent immunohistochemical studies have shown Topo II α to be a reliable indicator of cell proliferation in tumors, such as breast, ovarian, and bladder carcinomas, vulvar squamous lesions and oral lichen planus [42-46]. Here, Topo II α was immunohistochemically assessed in oral epithelial dysplasias, and carcinomas. Topo II α is an essential cellular enzyme that functions in the segregation of newly replicated chromosome pairs, in chromosome condensation, and in altering DNA superhelicity [47]. Because the expression of Topo II α isoform increases during the late S phase, decreases at the end of the M phase, and is dramatically reduced in the G1/G0 phase of the cell cycle, [48] an anti-Topo II α antibody labels cells in the S, G2, and M phases of the cell cycle [47]. The immunohistochemical method for the in situ determination of Topo II α has been validated extensively and shown to reflect closely the exact enzyme activity in formalin fixed paraffin wax embedded human tissues [49]. On the contrary, techniques such as western or northern blotting are averaging techniques and do not assess the amount of Topo II α in one particular neoplastic cell.

In general, the biological behavior of human malignancies is influenced by two major biological features of cancer cells: abnormal proliferation and the potential to invade and metastasise, which occasionally correlate with each other [50].

In the present study, the intense positive nuclear immunostaining expression of Topo II α was observed in the basal and parabasal layers (stratum germnativum layer) of nearly all normal oral epithelium regions while the granular and corneum cell layer showed less and variable degree of positive nuclear immunostaining. This is in agreement with the results of Liu et al., [51] who studied the proliferative rate in intra oral sites and found that basal cell layer and parabasal layer (stratum germnativum cell layer) show high proliferative activity and no proliferative activity was seen above the parabasal layer (superficial layer). In this field Holden et al [52], Hellemans et al [53] and Sandri et al [54] explained that the distribution of Topo II α in normal human tissue was restricted to sites known to harbor actively dividing cells. Also Truly et al [55] found that Topo II α is detected in proliferating compartment of all normal tissues, as would be expected with a cell division-specific role, as mitotic chromosomes segregation and/or condensation.

Hotchin, Gandarillas and Watt [56] found that the proliferation of keratinocyte is primarily restricted to the basal layer. When keratinocytes divide, down-regulate cell surface integrins to lose adhesiveness, leave the basal layer, exit the cell cycle and undergo a program of terminal maturation as they move through the suprabasal layers to the tissue surface. During this journey, the keratinocytes undergo a series of physiological and morphological changes that terminate by production of dead, flattened enucleated squames that are shed and replenished by differentiating keratinocytes.

The intense nuclear and cytoplasmic immunoexpression of Topo II α is observed in basal and parabasal layer in studied epithelial tissues, although Earnshaw et al [57] revealed that Topo II α has been shown to be a component of two highly insoluble protein fractions from chromosomes and nuclei, so these observation suggested that Topo II α might be an integral structure of the nucleus.

The Topo II α in the studied tissues gives variable reaction among different human oral tissues. The values of Topo II α between studied cases cleared that it can be ordered the oral tissues from highest to lowest immunoexpression as follow lip, gingival inferior surface of tongue and hard palate. The difference in immunoexpression of Topo II α among different normal oral human tissues may be explained by different opinion of many authors, Kellett, Hume and Potten, [58], who studied the proliferation rates and DNA synthesis in a continuous strip of epithelium from the gingival sulcus to the ventral surface of the tongue of mice and they found that the peak of proliferation and DNA synthesis in floor of mouth (lining mucosa) is higher followed by attached gingiva then free gingival epithelium. They concluded that there are various aspects of the distribution of DNA synthesis and proliferation in relation to type and site of the studied cells.

The epithelial covering of the labial mucous membrane in the present study showed highest nuclear immunoexpression of Topo II α among different other oral tissue. This is coincided with Thomson et al [59] who studied the difference in mitotic index between human oral mucosa tissues and found that there was a significant difference between anatomical sites, with higher mitotic count observed in buccal mucosa than mandibular gingiva. Meanwhile Nagase et al [60] stated that the turnover time is the estimated time needed to replace all cells in the epithelium and is derived from knowledge of time taken for a certain cell to divided and pass through the entire epithelium. They added that the nonkeratinized epithelium turnover faster than keratinized gingival epithelium and in general the turnover time for skin needs 75 days, for cheek 25 days and for gingiva 41–57 days. They concluded that there are regional difference in a pattern of epithelial proliferation and maturation associated with different turnover rates.

In the present study the keratinized epithelium of the gingival tissues showed higher expression to Topo II α than keratinized epithelium of hard palate in spite of that both tissues are masticatory mucosa, this may be due to the difference in type of keratinization as Eckert, Crish and Robinson [61] stated that in the oral cavity, Orthokeratinized epithelium similar to that in skin is seen in the hard palate, whereas other regions are either parakeratinized (gingiva) and parakeratinized epithelium divide faster than Orthokeratinized epithelium.

Also in the present study the epithelium of the lining mucosa of the lip showed higher immunostaining than that found in inferior surface of tongue this may be explained by Burns et al [62] explained that there are several possible explanations the most obvious explanation relates to the possibility that gene expression may be regulated differently. And the regional differences in gene expression could have many possible consequences. For example, differential responsiveness to hormones and growth factors and in their receptor, as there are differences in alpha adrenergic receptor number, might in turn influence the time of appearance and activity of enzymes and regulators involved in replication and differentiation.

In more details Philippe et al [63] suggested that the differences in behavior of epithelial tissue from various regions may reflect on site intrinsic characteristics to cells, or cell clones rather than to external factor. They explained that the disparate properties intrinsic to cells might due to regionally different modes of cellular communication including direct transmission of chemical messengers between contiguous cells either by paracrine and autocrine mechanisms. They added that regional differences in adipose tissue growth and distribution reflect intrinsic cellular properties, rather than locational disparities or external influences as blood or nerve supply, or ambient temperature.

In the current work the epithelial lining of oral mucous membrane of young group reveal intense immunoexpression to Topo II α than old group of different human oral tissues population, they are highly significant P < 0.01. This is in agreement with Stewart et al [64], who demonstrated that the cells associated with aging, is decreased metabolic efficiency (reduced growth rate), reduced off spring production, and with increased chance of death. Cardelli et al. [65] found that a progressive decrease of proliferation rate was found during both physiologic aging in vivo and induced aging in vitro.

In the other hand, Celenligil-Nazliel et al [66] found that there is no significant difference was observed between different age group with respect to proliferative activity in healthy gingiva. Age-related changes in proliferative activity in human gingival epithelium are uncertain. All the tissue sections contained positive staining cells for PCNA in the gingival epithelium. Although PCNA expression was observed both in the basal and suprabasal layers, it was more prominent in the suprabasal layers. But in inflamed gingiva was significantly higher in the older group. The proliferative activity was found to be increased with aging.

As well as Evans, Galasko and Ward, [67] studied the effect of age on growth of bone and found that the ability of individual cells to divide and to perform specific synthetic activities namely, total protein, osteocalcin, and alkaline phosphatase synthesis, did not show any change with increasing donor age. These results suggest that while the ability of individual cells to divide and to perform is unimpaired with increasing age, other subtler changes may occur, leading to a decrease in the bone's osteogenic capacity.

In our study, we also found that the Topo II-α index increased with progression from OED to OSCC, presumably reflecting the increase in the number of cycling tumor cells in invasive carcinomas.

Topo II-α was related to the clinicopathological parameters and no significant correlation was found between Topo II-α expression and patient's sex and tumor site. This finding was in agreement with previous reports on oral squamous cell carcinoma [68].

With regard to the age of OSCCs. High Topo II-α expression was more frequently detected among young age group of OSCCs and the correlation of high Topo II-α expression and age was statistically significant. In contrast, Stathopoulos et al [69] found that there was no statistically significant correlation between them.

With regard to the degree of tumour differentiation, the incidence of high Topo II-α immunopositivity was significantly greater in poorly differentiated SCCs. This strong correlation is in agreement with one previous study in head and neck squamous cell carcinomas [68] as well as in other malignancies [49,52]. In malignant cells, overexpression of the Topo II-α protein might reflect not only the proliferative advantage of these cells, but also qualitative alterations caused by malignant transformation and dedifferentiation. [47] In a recent study, treatment of the hepatoma cell line Hep3B with retinoids(which can induce cell differentiation) appeared to have a direct effect on the Topo II-α gene promoter because it greatly reduced both the steady state amount of mRNA and the transcription rate of the Topo II-α gene[70]. This Topo II-α activity increased as tumor differentiation was decreased and primary tumor size (T), regional lymph node metastasis (N), and disease stage advanced, resulting in poor prognosis. This finding was in agreement with Segawa et al [68] who carried out a study on oral squamous cell carcinomas. On the other hand, Stathopoulos et al [69] reported differently and stated that Topo II-α index of head and neck squamous cell carcinoma did not correlate with clinicopathological parameters such as histologic type, lymph node metastsasis, and tumor site. In addition, we found that there was significant relationship between Topo II-α and EBV expression in primary lesions was higher in cases with lymph node metastasis than in those without lymph node metastasis. As EBV expression increased, the Topo II-α LI significantly increased. These findings coincided with those of the present study. Therefore, these two enzyme activities may be valuable diagnostic and prognostic indices in oral carcinoma.

As regards the two evaluated markers (Topo II-α and EBV), they were not significantly related to each other in OSCCs. However, increased expression of EBV correlated significantly with elevated Topo II-α in OEDs. In addition to, the expression of EBV was increased in the progression from of OEDs to OSCCs, with elevation of Topo II-α LI. These findings strongly indicate that EBV may contribute to malignant transformation and tumor growth. This result was in agreement with Shimakage [38].

## Conclusion

1-Topo II α can be used as a valuable biomarker to estimate proliferative activity in various sites of normal oral mucous membrane. As there are direct relation between the level of immunoexpression of it and the degree of proliferation of examined tissue. This can give alert about the proliferation state of normal cell or abnormal cell.

2-Increased expression of Topo II α expression in almost all examined cases is related to the histological grade and the stage of the disease, and denotes aggressive biological behavior. Accordingly, Topo II α expression may be helpful in identifying those oral squamous cell carcinomas with higher malignant potential, and could be used for staging evaluation and predicting prognosis in premalignancy and oral carcinomas.

3-Increased expression of EBV protein correlated with elevated Topo II-∝ LI, indicating that EBV may contribute to malignant transformation and tumor growth.

4-Age does not seem to be a risk factor for EBV infection. Increased age did not enhance EBV prevalence.

Further studies are needed to reveal the role played by EBV in carcinogensis of squamous cell carcinoma and to study the impact of EBV infection on cellular immune response to oral cancer. Furthermore, the etiologic role of EBV in OSCC and OED needs to be examined in a prospective follow-up study.

## Competing interests

The authors declare that they have no competing interests.

## Authors' contributions

AS and MZ conceived of the study, carrying out immunohistochemistry, interpretation of results, performed the statistical analysis and writing of the manuscript. MW and AA participated in the histopathological diagnosis, and editing of the manuscript. SA and MM collecting samples, selecting cases, review the patients clinical data and editing of the manuscript. All authors read and approved the final manuscript.
